# Thinning of Poly(methyl methacrylate) and Poly(vinyl chloride) Thin Films Induced by High-Energy Ions of Different Stopping Powers

**DOI:** 10.3390/polym15234471

**Published:** 2023-11-21

**Authors:** Raquel Thomaz, Yvette Ngono-Ravache, Daniel Severin, Christina Trautmann, Ricardo M. Papaléo

**Affiliations:** 1 Interdisciplinary Center of Nanoscience and Micro-Nanotechnology, School of Technology, Pontifical Catholic University of Rio Grande do Sul, Porto Alegre 90619-900, RS, Brazil; 2 Ion Implantation Laboratory, Institute of Physics, Federal University of Rio Grande do Sul, Porto Alegre 91501-970, RS, Brazil; 3 CIMAP, UMR CEA-CNRS-ENSICAEN-UCBN, BP 5133, CEDEX 5, F-14070 Caen, France; 4 Materials Research, GSI Helmholtz Centre, 64291 Darmstadt, Germany; 5 Department of Materials- and Geosciences, Technische Universität Darmstadt, 64287 Darmstadt, Germany

**Keywords:** thinning, polymers, ion irradiation

## Abstract

Ion bombardment is an important tool of materials processing, but usually leads to erosion of the surface and significant thickness reductions when thin layers are used. The growing use of polymer thin films in a variety of applications, from coatings and membranes to biomedical and electronic devices, calls for a deeper understanding of the thinning process induced by energetic ions espe-cially for very thin films. Here, thinning and surface morphology changes induced by high-energy ion bombardment in PMMA and PVC thin films were investigated, focusing on the role of the initial thickness of the films and the stopping power of the ions. We used thin films with initial thicknesses varying from 13 to 800 nm, and light and heavy ions as projectiles in the energy range of 2–2000 MeV, where the electronic stopping dominates. Thickness reductions as a function of fluence were monitored and thinning cross sections were extracted from curves. A supralinear scaling between the thinning cross sections and the electronic stopping power of the beams was observed, with a much enhanced thinning efficiency for the swift heavy ions. The scaling with the stopping power dE/dx is almost independent of the initial thickness of the films. At intermediate and large fluences, changes in the physicochemical properties of the irradiated polymers may modulate and decelerate the thinning process of the remaining film. The importance of this secondary process depends on the stopping power and the balance between erosion and the chemical transformations induced by the beam. We also observe a trend for the thinning efficiency to become larger in very thin films. Depending on the type of beam and polymer, this effect is more or less pronounced. PMMA films irradiated with 2 MeV H^+^ show the most systematic correlation between initial thickness and thinning cross sections, while in PVC films the initial thickness plays a minor role for all investigated beams.

## 1. Introduction

It is well known that ion irradiation with energetic ions can induce irreversible changes in the structure of polymers and other organic materials, resulting in profound alterations in their mechanical, optical, and electrical properties [1,2,3,4,5]. One of the basic effects of ion bombardment is a thickness reduction that is usually connected to the direct erosion (sputtering) of the surface by the beam. For radiolytic materials such as polymers, the release of volatile fragments and/or the subsequent relaxation and compaction of the films (or a combination of both processes) also influence the process [1,2,3,4,5,6]. For example, the thickness decrease in irradiated poly(methyl methacrylate), PMMA, has been attributed to the scission of the pendent groups of the monomer followed by the diffusion and release of fragments and gas molecules. A good correlation was found between outgassing, weight loss, and thickness reduction of irradiated PMMA. Scission of the backbone also occurs [7,8], increasing the macromolecular mobility and favoring the relaxation of the chains and compaction of the films [9,10,11,12,13]). For polyvinyl chloride (PVC), irradiation gives rise to an important loss of mass via the emission of HCl and H_2_ [14,15,16,17]. In particular, the Cl concentration decreases quickly during irradiation [18,19,20], accompanied by a decrease in thickness [21]. At medium to high fluences, thinning is a complex phenomenon, involving also changes in the chemical structure and physical properties. Under extensive ion bombardment and high deposited energy densities, polymer films turn into some form of hydrogenated carbon layer, and thinning at that stage has very little to do with the original polymer behavior [22].

In spite of past efforts, the available experimental data on the thinning process is still limited and mainly connected to the low kinetic energy regime, used in plasma or ion beam treatment (0.1–10 keV) [23,24]. Systematic investigations on the thinning process, especially those intercomparing different energy deposition regimes or the effect of the initial thickness of the films are still scarce. At the same time, the growing use of polymer thin films in a variety of applications, from coatings and membranes to biomedical and electronic devices, calls for a deeper understanding of the thinning process, especially for very thin films. Here, we report on a study where the effect of the initial thickness of the polymer layer on the thinning process is investigated. We investigated two polymers (PMMA and PVC) with initial thicknesses varying from 13 to 800 nm, and, as projectiles, light and heavy ions in the energy range of 2–2000 MeV, where the electronic stopping (dE/dx)_e_ dominates. Thinning and other morphological changes in the films (roughness, porosity) were monitored offline by scanning force microscopy (SFM). We show that thickness reductions in PMMA induced by light ions are dependent on the initial layer thickness $h_{0}$, while for swift heavy ions irradiation, the thinning rate is to a certain extent independent of the initial thickness $h_{0}$. On the other hand, the thinning of PVC films was almost independent of the film thickness for all the investigated beams.

## 2. Materials and Methods

**Figure 1:**
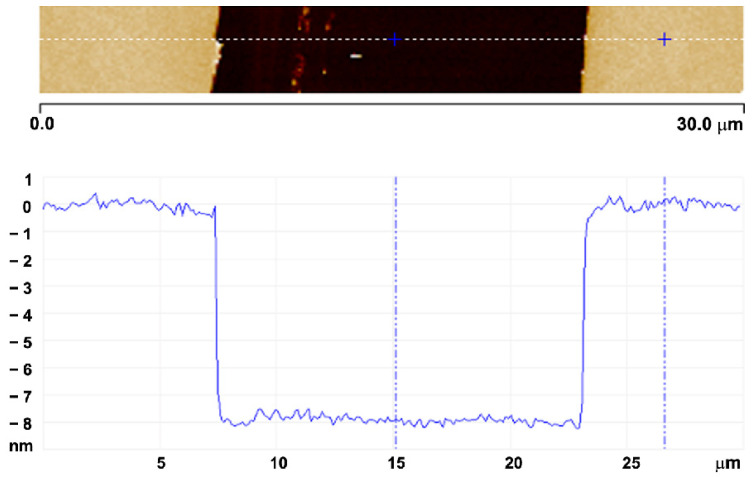
SFM image showing a region around a micro-scratch created on an 8 nm thick film and its corresponding height section.

Poly(methyl methacrylate) (PMMA, Mw ~120,000 u) and polyvinyl chloride (PVC, Mw ~120,000 u) powders were purchased from Sigma-Aldrich Brasil LTDA, SP, Brazil. PMMA was diluted in anisole and PVC in cyclohexanone, at different concentrations from 5 g/L to 80 g/L. Thin films of both polymers were spun from the solutions onto Si substrates at 3000 rpm for 45 s, using a Laurell WS-400BZ-6NPP/LITE spin coater from Laurell Technologies Corporation, Lansdale, PA, USA. After deposition, the samples were annealed on a hot plate at 70 °C for 60 min to allow relaxation of the films and elimination of residual solvents. SFM was used for measuring the thickness of the layers before and after ion beam irradiation and for the investigation of the film surface morphology. SFM measurements were performed with a Bruker Dimension Icon instrument from Bruker Corp (Billerica, MA, USA), in the peak force mode in the air using silicon nitride probes (Bruker ScanAsyst-Air) with a nominal tip radius of 2 nm and a resonant frequency of 70 kHz. The images were recorded at a scan frequency of 1–2 Hz with 256 scan lines in 1 µm scans. Thickness measurements using SFM were obtained from the depth of a trench made on the films with a sharp scalpel as illustrated in Figure 1. This technique is reliable for thicknesses down to 2 nm with uncertainties below ±0.6 nm. The initial thickness $h_{0}$ of the deposited films ranged from 13 to 800 nm, depending on the concentration of the polymer solution. The root-mean-square roughness ($R_{RMS})$ was almost independent of the film thickness and had a typical value of around 0.25 nm. To measure the thickness of the pristine films, other techniques such as ellipsometry and resonant Rutherford backscattering spectrometry (RRBS) through the reaction ^12^C(α, α′)^12^C at 4.285 MeV were also employed, yielding results in good agreement with the SFM measurements.

The samples were bombarded by 2 MeV H^+^ and 17 MeV Au^7+^ at the 3 MV Tandetron (at Porto Alegre, Brazil), 12 MeV C^4+^ ions (at GANIL, Caen, France), and 2.1 GeV Bi ions (at the GSI-UNILAC, Darmstadt, Germany). The range of all ion beams was significantly larger than the thickness of the polymer films. The fluence range, the electronic and nuclear stopping powers, and the range for each ion beam are given in Table 1. Electronic and nuclear stopping powers and ranges were extracted from the simulation code SRIM 2012 [25].

## 3. Results

### 3.1. PMMA Thin Films

**Figure 2:**
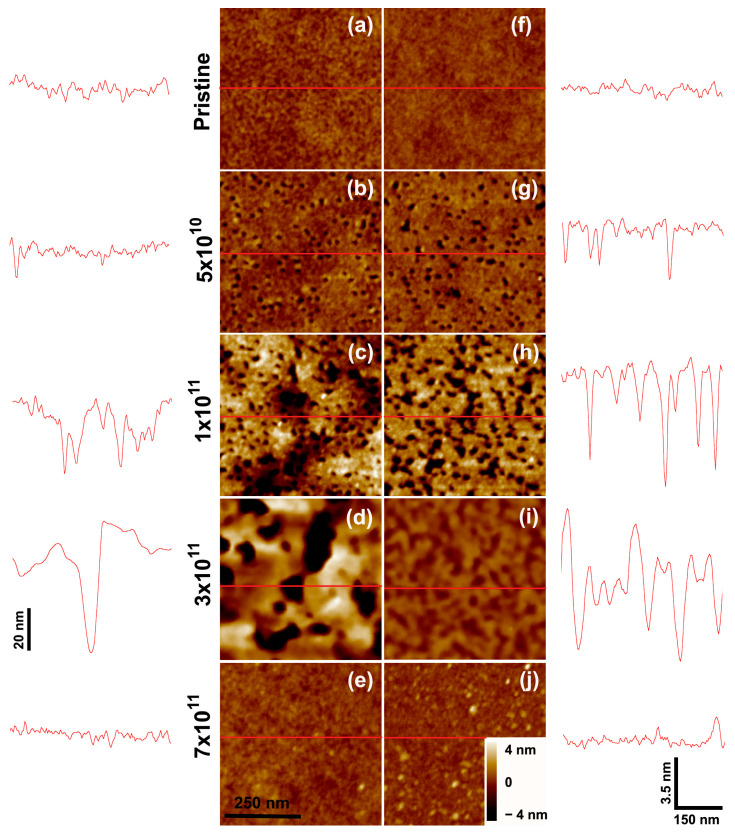
SFM images of (**a**–**e**) 100 nm and (**f**–**j**) 20 nm thick PMMA films bombarded by 2.1 GeV Bi ions at various fluences: (**a**,**f**) pristine sample surfaces; (**b**,**g**) ϕ = 5 × 10^10^ ions/cm^2^; (**c**,**h**) ϕ = 10^11^ ions/cm^2^; (**d**,**i**) ϕ = 3 × 10^11^ ions/cm^2^; (**e**,**j**) 7 × 10^11^ ions/cm^2^. The traces to the sides depict scan lines along the path indicated in red on the images. The horizontal and vertical bars at (**j**) give the lateral and height scales for the line profiles, except in (**d**), where the depth scale is different. In the SFM images, the color scale corresponds to height variations from −4 nm to +4 nm, except in image (**d**) where color contrast spans −25 nm to +25 nm.

**Figure 3:**
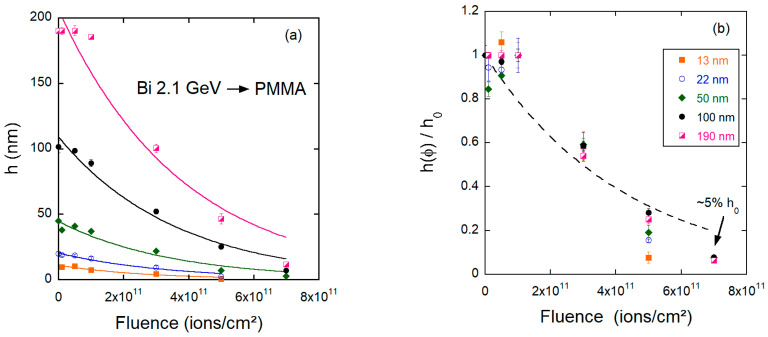
(**a**) Absolute and (**b**) relative values of the PMMA film thickness as a function of fluence for samples bombarded by 2.1 GeV Bi ions.

Figure 2 shows SFM images (together with selected scan line profiles) of PMMA films irradiated with 2.1 GeV Bi ions (equilibrium charge state distribution) at different fluences. The two columns show the respective data for 100 nm and 20 nm thick films and allow visualization of morphological and roughness changes introduced by the irradiation. The evolution of the film thickness as a function of irradiation fluence for these samples is depicted in Figure 3. Although a steady decrease in film thickness with increasing fluence is observed, the morphology of the surface goes through distinct stages as the irradiation progresses. At low fluences (up to ϕ ~5 × 10^10^ ions/cm^2^), craters induced by individual ion impacts can be distinguished on the initially smooth PMMA surface (Figure 2b,g). The number of craters seen in the SFM images is close to the nominal fluence. Cross-sectional profiles taken from these images show that the craters are about 3.5 nm deep and ~18 nm wide, consistent with values reported in previous works. For very thin films (usually below 10 nm), the crater volume of individual ion impacts starts to decrease [26], but this effect is small at the thickness range explored here.

In the low fluence regime, the surface porosity of the films increases, but the thickness remains essentially the same. This regime is seen as a small plateau in the *h* versus ϕ curves of Figure 3a. Larger fluences (10^11^ ions/cm^2^) lead to overlapping craters, where the counting of individual craters is no longer reliable (Figure 2c,h). Additionally, the cross-sectional profiles reveal not only wider but deeper holes on the surfaces. This causes the roughness $R_{RMS}$ to rise from 0.25 nm to about 2 nm for the thickest film investigated. The progressing erosion of material induces a thickness reduction, yet this is a relatively small effect (Figure 3a). At a fluence of 3 × 10^11^ ions/cm^2^, the roughness of the film peaks. The surface holes are large and irregular, and their depth becomes comparable to the remaining film thickness (about half of its initial value, Figure 3b). Thus, in this regime, a membrane-like morphology is reached. For even larger fluences, the layers continue to thin down. At 7 × 10^11^ ions/cm^2^, the remaining thickness is only 5% of its initial value. The thinning process in the high fluence regime also leads to a drastic smoothing of the surface, as shown in (Figure 2e,j).

When plotting the thickness after bombardment $h\left(\phi \right)$ scaled by the initial thickness *h*_0_ as a function of fluence (Figure 3b), the data follow roughly a master curve, suggesting that for swift heavy ions, the thinning rate is to a certain extent independent of the initial thickness $h_{0}$. Ignoring the plateau regime and assuming that the thinning process can be described by an exponential (Poisson) law of the type $h\left(\phi \right)=h_{0}e^{-\sigma \phi }$, a thinning cross-section σ can be extracted. The obtained values are given in Table 2. Cross sections are similar for the different $h_{0}$. There is an increase in σ for the thinnest film, but this is within the uncertainties of the measurements. The average cross section (among all thicknesses analyzed) for 2.1 GeV Bi ions is $\sigma =(2.3\pm 0.5)\times 10^{-12}\;$cm^2^.

**Figure 4:**
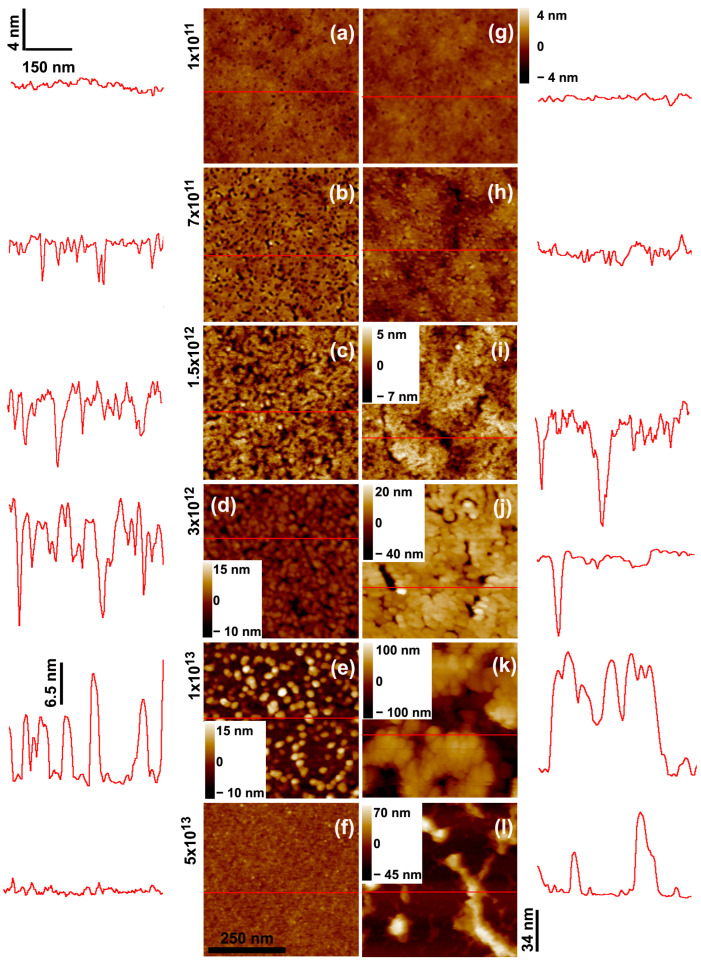
SFM images of (**a**–**f**) 25 nm and (**g**–**l**) 200 nm thick PMMA films deposited on Si wafers and bombarded by 17 MeV Au^7+^ ions with fluences of 1 × 10^11^ ions/cm^2^ (**a**,**g**); 7 × 10^11^ ions/cm^2^ (**b**,**h**); 1.5 × 10^12^ ions/cm^2^ (**c**,**i**); 3 × 10^12^ ions/cm^2^ (**d**,**j**); 1 × 10^13^ ions/cm^2^ (**e**,**k**); and 3 × 10^13^ ions/cm^2^ (**f**,**l**). The traces to the sides depict scan lines along the path indicated in red on the images. The horizontal and vertical bars at (**a**) give the lateral and height scales for the line profiles, except for the height scale in (**e**) which is 6.5 nm, and (**j**–**l**) which is 34 nm. In the SFM images, the color scale corresponds to height variations from −4 nm to +4 nm, when the color contrast is not shown in the images.

**Figure 5:**
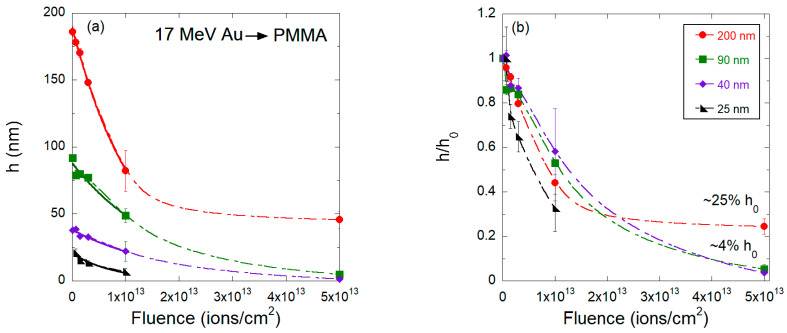
(**a**) Absolute and (**b**) relative values of the PMMA film thickness as a function of fluence for samples bombarded by 17 MeV Au ions. The solid lines are exponential fittings for fluences up 5 × 10^13^ ions/cm^2^ from which the thinning cross-sections were extracted, and the dashed line are guides to the eyes.

Next, we discuss the thinning process induced by 17 MeV Au^7+^ ions. Figure 4 presents SFM images of 25 and 200 nm thick PMMA films irradiated at fluences up to 5 × 10^13^ ions/cm^2^, while Figure 5 shows the curve of film thicknesses as a function of fluence. Although there are some similarities to the thinning process of 2.1 GeV Bi ions, the initial stages for the gold beam are somewhat different. For example, no individual craters induced by 17 MeV Au ion impacts can be identified in the low fluence regime (10^9^–10^10^ ions/cm^2^). Starting at ϕ = 10^11^ ions/cm^2^, surface holes appear which are shallower than those produced by Bi ions. Their areal density is smaller than the ion fluence, suggesting that they do not originate from individual ion impacts but from collective effects. At intermediate fluences, the overall film surface topography is less degraded compared to the Bi beam. On the other hand, the roughness of the irradiated films also initially increases, peaks at intermediate fluences (ϕ = 1 × 10^13^ ions/cm^2^, Figure 4e,k), and decreases for larger fluences. The flattening of the surface at high fluences occurs in the 25 nm thick sample, but not in the thick samples, which in contrast have larger islands of degraded material (Figure 4l).

Figure 5 reveals the thinning as a function of the fluence of 17 MeV Au ions. Compared to Bi ions, both the thinning rate and the residual thickness of the PMMA films depend more strongly on the initial thickness *h*_0_. For thick films ($h_{0}$ = 800, 400, and 200 nm) irradiated by 17 MeV Au ions of maximum fluence, the residual thickness is about 25% of its initial value. Moreover, for such thick films, the thinning process is not well-described by a single rate (or cross section) because at about ϕ = 1 × 10^13^ ions/cm^2^, the thinning rate (discussed below) decreases significantly, most possibly due to chemical modifications induced by the irradiation, such as crosslinks and carbonization [27]. In contrast, thin films ($h_{0}$ = 90, 40, and 20 nm) are thinned down to 4% or less of $h_{0}$, and the thinning process follows a single rate mechanism.

For the purpose of comparison with the other beams, the thinning cross sections for the 17 MeV Au ions reported in Table 2 were extracted, excluding the point at ϕ = 5 × 10^13^ ions/cm^2^ (solid lines in Figure 5a). This is to avoid the distortions that a simple exponential decay would introduce in the case of the thick films. For most of the different $h_{0}$, the cross sections are close to $(6.2\pm 2.0)\times 10^{-14}$ cm^2^. Only for the thinnest films ($h_{0}$ = 25 nm) is the decay rate clearly larger, at around 12.5 × 10^−14^ cm^−2^.

**Figure 6:**
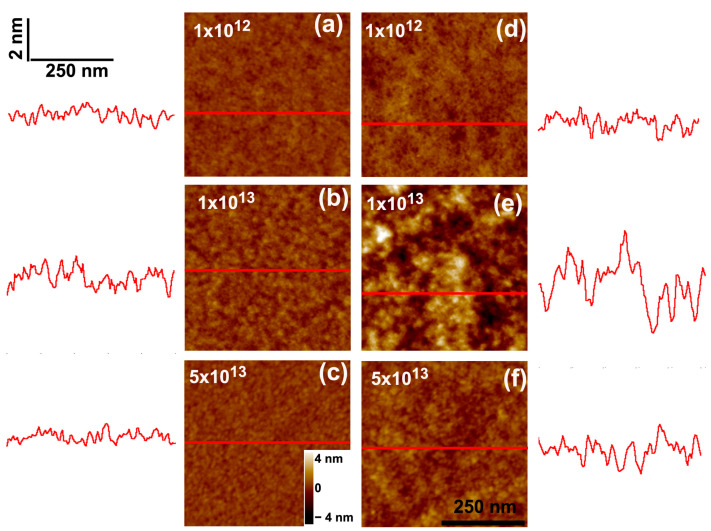
SFM images of (**a**–**c**) 6 nm and (**d**–**f**) 95 nm thick PMMA films deposited on Si wafers bombarded by 12 MeV C^4+^ ions with fluences up to 5 × 10^13^ ions/cm^2^. The traces to the sides depict scan lines along the path indicated in red on the images. The horizontal and vertical bars at (**a**) give the lateral and height scales for the line profiles. In the SFM images, the color scale corresponds to height variations from −4 nm to +4 nm.

**Figure 7:**
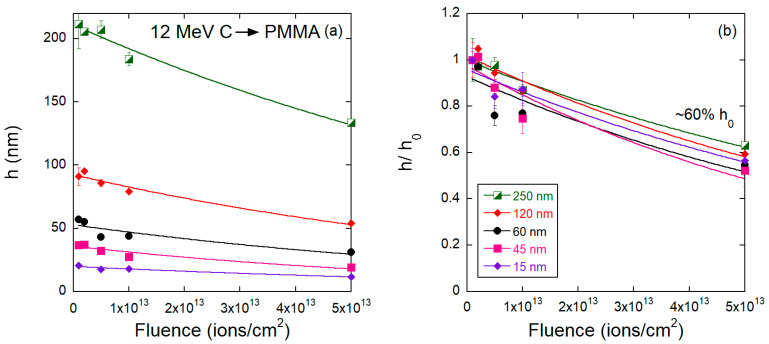
(**a**) Absolute and (**b**) relative values of the PMMA film thickness as a function of fluence for samples bombarded by 12 MeV C^+4^ ions.

**Figure 8:**
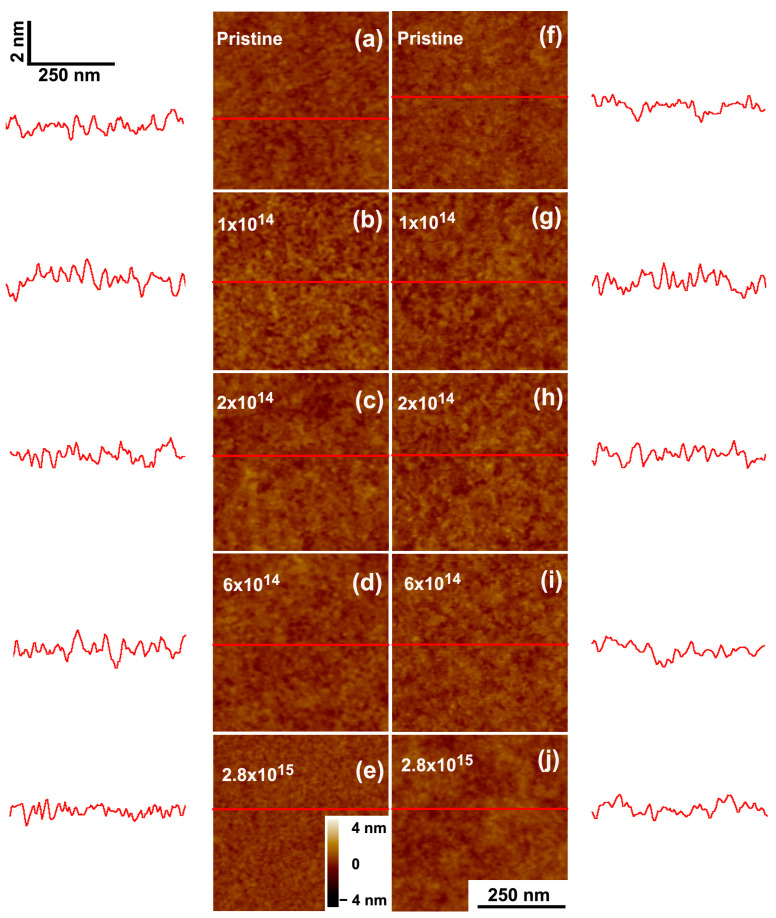
SFM images of PMMA films irradiated by 2 MeV H^+^ of two initial thicknesses: (**a**–**e**) 14 nm and (**f**–**j**) 116 nm; (**a**,**f**) as-deposited films; (**b**,**g**) 1 × 10^14^ ions/cm^2^; (**c**,**h**) 2 × 10^14^ ions/cm^2^; (**d**,**i**) 6 × 10^14^ ions/cm^2^; (**e**,**j**) 2.8 × 10^15^ ions/cm^2^. The traces to the sides depict scan lines along the path indicated in red on the images. The horizontal and vertical bars at (**a**) give the lateral and height scales for the line profiles. In the SFM images, the color scale corresponds to height variations from −4 nm to +4 nm.

**Figure 9:**
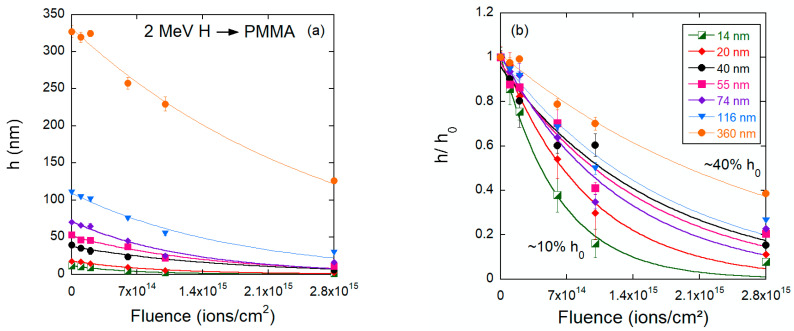
(**a**) Absolute and (**b**) relative values of the PMMA film thickness as a function of fluence for samples bombarded with 2 MeV H^+^.

The results of the thinning process induced by the low stopping power beams (12 MeV C and 2 MeV H) are presented in Figure 6, Figure 7, Figure 8 and Figure 9. The response is different from that induced by the heavier ions. The light ions induce neither impact craters nor the subsequent increase in surface porosity seen for Bi or Au irradiations, and the surfaces of the carbon and proton irradiated films show minimal roughening. Yet, the thinning induced by 2 MeV H^+^ and 12 MeV C ions in PMMA differs. For the carbon beam (Figure 7), the residual thickness after the maximum fluence $h\left(\phi_{max}\right)\;$is about 0.6 $h_{0},$ and the thinning cross sections are also similar for all initial thicknesses (~10^−14^ cm^2^). For the proton irradiated samples (Figure 9), on the other hand, both the thinning cross section and the residual thickness at maximum fluence are clearly dependent on the initial thickness. The cross sections systematically increase (from 3.4 × 10^−16^ cm^2^ to 16 × 10^−16^ cm^−2^), (Table 2) and the residual thickness decreases (from about 0.4 *h*_0_ to 0.1 *h*_0_).

### 3.2. PVC Thin Films

**Figure 10:**
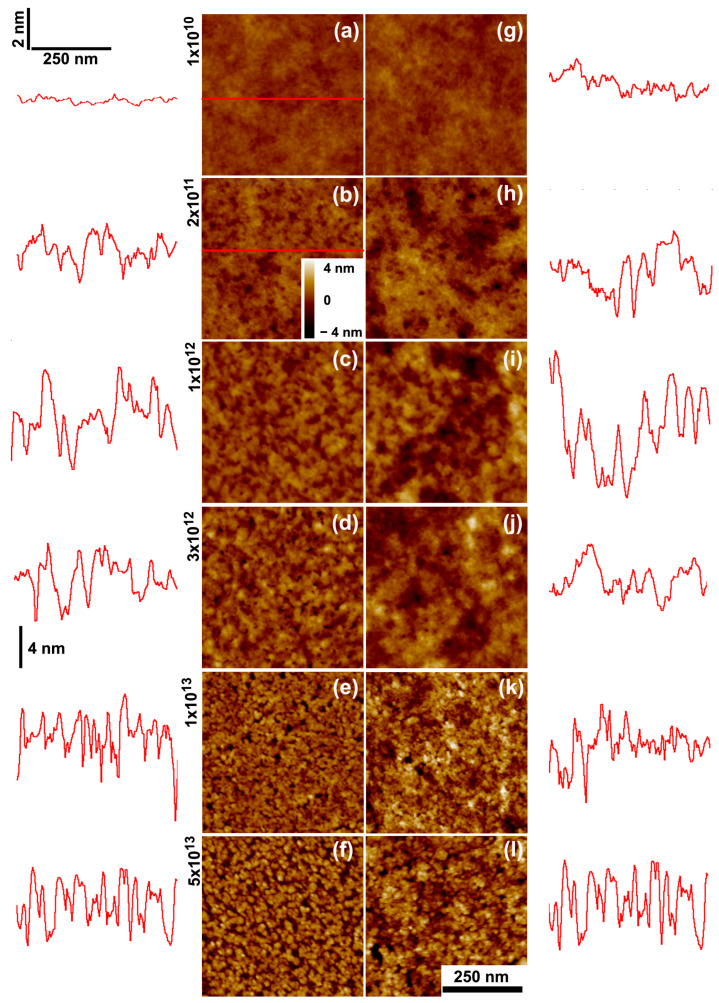
SFM images of (**a**–**f**) 25 nm thick and (**g**–**l**) 290 nm thick PVC films deposited on Si wafers and bombarded by 16 MeV Au^7+^ ions with fluences up to 5 × 10^13^ ions/cm^2^. The color contrast covers height variation of −4 to +4 nm in images (**a**,**b**,**g**,**h**), and −8 to 8 nm in images (**d**–**f**) and (**j**–**l**). The traces to the sides depict scan lines along the path indicated in red on the images. The horizontal and vertical bars at (**a**) give the lateral and height scales for the line profiles for samples irradiated with fluences up to 1 × 10^12^ ions/cm^2^ and the vertical bar for larger fluences is given in (**d**).

**Figure 11:**
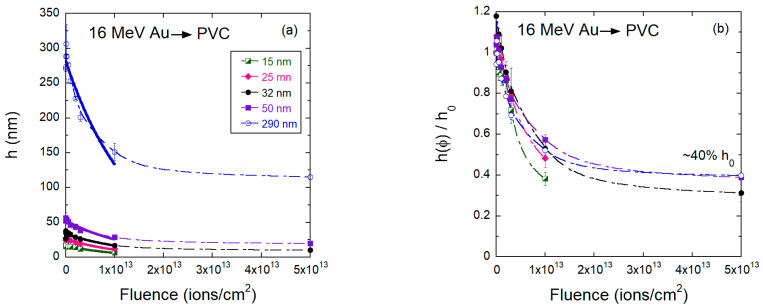
(**a**) Absolute and (**b**) relative values for the PVC film thickness as a function of fluence for samples bombarded by Au 16 MeV ions. The solid lines are exponential fittings for fluences up 1 × 10^13^ ions/cm^2^ from which the thinning cross-sections were extracted, and the dashed line are guides to the eyes.

**Figure 12:**
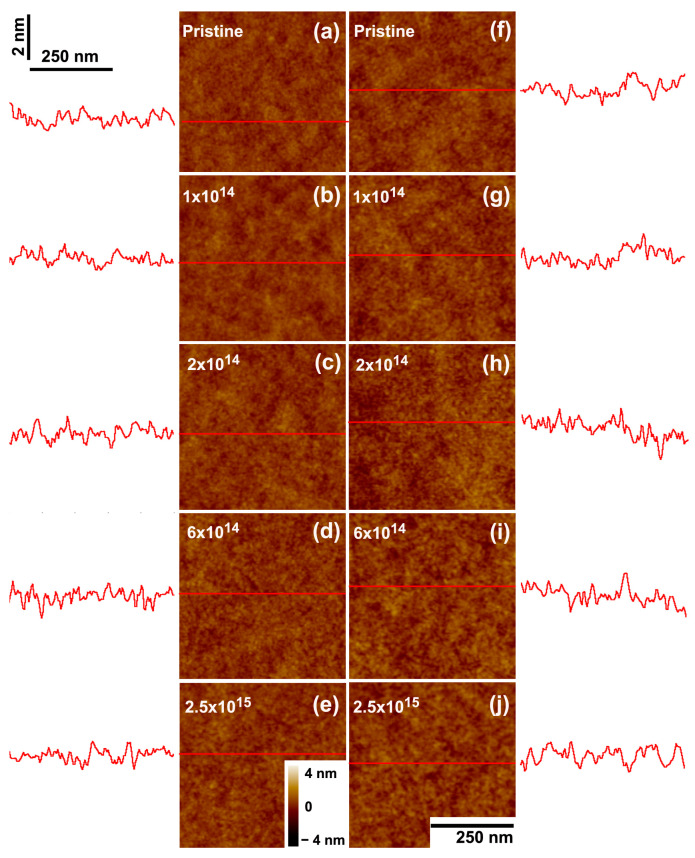
Topography of PVC (**a**–**e**) 15 nm and (**f**–**j**) 90 nm thick films. SFM images of films as-deposited (**a**,**f**) and bombarded by 2 MeV H^+^ at 1 × 10^14^ ions/cm^2^ (**b**,**g**); 2 × 10^14^ ions/cm^2^ (**c**,**h**); 6 × 10^14^ ions/cm^2^ (**d**,**i**); and 2.8 × 10^15^ ions/cm^2^ (**e**,**j**). The traces at the sides depict scan lines along the path indicated in red on the images. The horizontal and vertical bars at (**a**) give the lateral and height scales for the line profiles. In the SFM images, the color scale corresponds to height variations from −4 nm to +4 nm.

**Figure 13:**
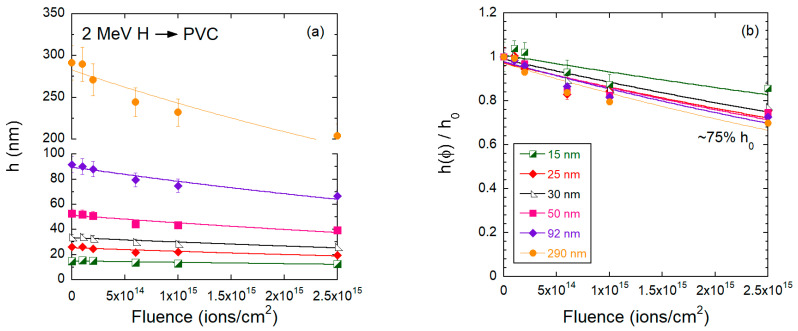
(**a**) Absolute and (**b**) relative values for the PVC film thickness as a function of the fluence for samples bombarded by 2 MeV H^+^ ions.

Irradiations of PVC films were only performed with the 2 MeV H^+^ and 16 MeV Au beams (Figure 10, Figure 11, Figure 12 and Figure 13). Figure 10 presents SFM images of 25 nm and 290 nm thick PVC films irradiated with 16 MeV Au^7+^ with fluences up to 5 × 10^13^ ions/cm^2^. As in the case of PMMA, no craters due to single ion impacts are seen on the irradiated PVC surface at low fluences (Figure 10a,g). At a fluence of 2 × 10^11^ ions/cm^2^, surface pores are visible, although not as pronounced as for PMMA (Figure 4). With increasing fluence, the surface of PVC becomes rougher and develops a regular topographical structure, but the morphology patterns are distinctly different from those of the heavily bombarded PMMA films, which are more irregular and grainier. The evolution of the PVC film thickness with increasing fluence is shown in Figure 11 for the 16 MeV Au beam. Similar to PMMA bombarded by gold ions, the decrease in the PVC thickness is not well described by a single rate. This is especially clear for the 290 nm thick films, where the thinning rate slows down considerably at fluences above 1 × 10^−13^ ions/cm^2^. Thus, we also adopted the criterion of considering only the low fluence data and fitting a simple exponential function to obtain the thinning cross sections. The extracted cross sections are presented in Table 3. They vary among the various initial thicknesses between 7–10 × 10^−14^ cm^−2^. Similar values were found for PMMA irradiated with 17 MeV Au (see Table 2). However, the residual thickness at maximum fluence is clearly larger for PVC films, indicating that the structure of the partially *degraded* PVC is more resistant to thinning than PMMA. This is consistent with facile dehydrochlorination of PVC when exposed to high-energy radiation [19,21] and leads to a more rapid carbonization process, as compared to PMMA. Under the bombardment by 2 MeV H^+^, the residual thickness at maximum fluence is larger for PVC than for PMMA. We assume that the structure of radiation-induced intermediate carbonization is different for the two polymers.

The proton irradiation does not induce any marked change in the surface topography of PVC films (Figure 12), similar to what was seen in PMMA. However, the thinning cross sections extracted for PVC bombarded by 2 MeV H^+^ (Figure 13 and Table 3) are substantially smaller than for PMMA, especially for the thinner films. In contrast to what is observed in PMMA, the thinning cross sections in PVC are roughly independent of the initial thickness of the films (Table 3).

## 4. Discussion

**Figure 14:**
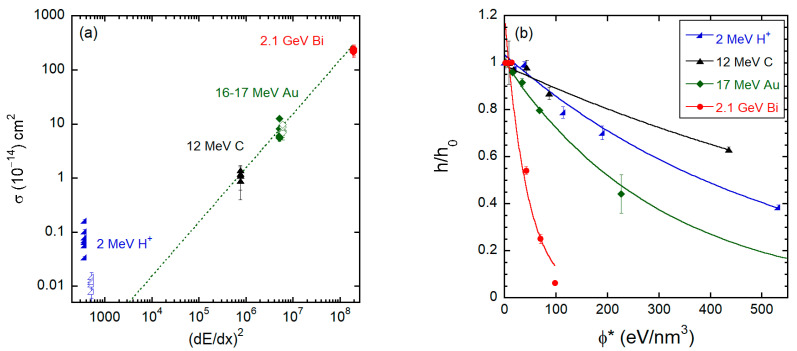
(**a**) Thinning cross sections as a function of the squared electronic stopping power for PMMA (solid symbols) and PVC (open symbols) for all beams and thicknesses investigated. (**b**) Relative thickness ${h/h}_{0}$ for thick PMMA films ($h_{0}$ = 190 − 360 nm) as a function of the mean deposited energy density $\phi^{*}=\phi \times {\left(dE/dx\right)}_{e}$.

The results presented in Section 3 reveal general trends in the thinning process common to all different beams, but also specificities that emerge either from the type of polymer, from the magnitude of the stopping power of the ions, or the initial thickness of the films. Regarding the overall efficiency of the thinning process, parametrized by the thinning cross sections, we found a quadratic correlation between the cross-section values and the stopping power of the beams. This is illustrated in Figure 14a, where the cross sections are displayed as a function of the electronic stopping power squared ${\left(dE/dx\right)}_{e}^{2}$ for both PMMA and PVC films. The group of points at the same ${\left(dE/dx\right)}_{e}^{2}$ corresponds to the cross sections for all different film thicknesses. The data from the 2 MeV H^+^ irradiation is clearly out of this scaling, most probably because of the very small contribution of sputtering erosion to the thinning process [28]. Figure 14b shows the evolution of the normalized thickness $h/h_{0}$ as a function of the mean deposited energy density ϕ* = *ϕ* × (dE/dx) for thick PMMA films ($h_{0}$ = 190 − 360 nm) irradiated by the different beams. The non-linear dependence on ϕ* is evident; 2.1 GeV Bi ions are by far the most efficient ions to thin down the polymers due to the very large sputtering yield. For Bi but also Au ions, this is evidenced by the craters formed on the surface of the samples. Crater volumes from individual swift heavy ion impacts in polymers increase strongly with electronic stopping power ${\left(dE/dx\right)}_{e}$ [29], reaching values around 500 nm^3^ or more for an individual ion such as 2.1 GeV Bi. This mechanism significantly enhances the thinning efficiency of this beam. Moreover, it has been shown that at very large electronic stopping powers the stoichiometry of irradiated PMMA (the O/C ratio) is closer to the pristine polymer than similar samples irradiated by a low dE/dx beam [28]. This indicates that under swift heavy ion bombardment, erosion is more stoichiometric and occurs at such a fast rate that the film is rapidly consumed, before significant structural modification takes place in the remaining material, as seen in [28] by X-ray photoelectron spectroscopy (XPS) measurements. This fact also partially explains why the thinning process for the Bi beam leads to a small residual thickness and is well described by a single exponential, while for the other beams a slowing down of the thinning rate is clearly seen at intermediate to large fluences, especially for thick samples.

We finally note that, although the sets of cross sections for thin and thick films display the same scaling with ${\left(dE/dx\right)}_{e}$, there is a clear trend for all tested beams that the thickness decrease is more efficient in very thin films. The dependence of the thinning cross section on the initial thickness is most evident in the PMMA samples irradiated by 2 MeV H^+^. The low sputtering yield, typically associated with light ions and low electronic stopping power [28], makes the thinning process more dependent on outgassing and compaction of the layers [14,19,21,27,30,31,32]. In thin films, the diffusion of volatile species towards the surface is facilitated resulting in enhanced thinning cross sections.

The actual scenario may be more complex, because of the eventual relaxation and compaction of the irradiated films (which may also be dependent on the initial thickness) and the type of carbon-enriched structure that is formed. The importance of the carbonization pathway is evident through the comparison of the thinning process in PVC and PMMA. For example, PVC films irradiated by 2 MeV H^+^ have thinning cross sections significantly smaller than those of PMMA and do not show the correlation between the initial thickness of the polymer and the thinning cross sections as observed in PMMA (Table 3). Moreover, for increasing fluence, the thinning rate of PVC slows down and the residual thickness at intermediate fluences is rather large. The single exponential scheme produces a poor global fit to the PVC data. As mentioned before, the process of dehydrochlorination caused by the fast diffusion of HCl and H_2_ [14,19,21] is a hallmark of irradiated PVC. The cross section for chlorine loss in PVC irradiated by 2 MeV H^+^ and probed by XPS measurements [20] is about 10 times larger than the one found here for the thinning process. At intermediate fluences, the remaining material has less than 20% of the initial chlorine content. Continued irradiation of the PVC residue triggers the formation of extended conjugated carbon unsaturated bonds, which may eventually stabilize the structure against further thinning. In polyethylene, for example, the formation of unsaturated bonds was shown to stabilize at high doses through electronic excitation transfers from the polymer to C=C [33,34]. For the 17 MeV Au beam, the change in the thinning rate at intermediate fluences is very pronounced in PVC, and also visible in PMMA. For PVC irradiated with both Au and proton beams, the residual thickness is larger than in PMMA films. Again, such differences can be linked to the efficient dichlorination of PVC and the polyene structures creation.

## 5. Conclusions

In this work, thinning and surface morphology changes induced by high-energy ion beams on PMMA and PVC thin films were investigated, focusing on the role of the initial thickness of the films, the polymer tendency to crosslink or to scission, and the stopping power of the ions. As the penetration of the ions is much larger than the film thickness, the modification induced by the beams is homogeneous along the film depth, contrary to common conditions of plasma or low-energy ion etching. Excluding the proton irradiations, the large data set suggests a quadratic dependence of the thinning cross sections on the electronic stopping power of the beam. Therefore, much enhanced thinning efficiency is generally expected for swift heavy ions. The scaling with dE/dx is roughly independent of the initial thickness of the films. At small to medium stopping powers, when sputtering is low, the irradiation induces significant chemical changes before substantial erosion occurs. Thus, physicochemical modifications of the irradiated polymers may decelerate the thinning process in the intermediate fluence regime. At large electronic stopping power, stoichiometric erosion is more dominant and occurs more rapidly than chemical transformations, yielding enhanced thickness reduction and smaller residual film thicknesses. Thus, the chemical structure of the monomeric unit can play a significant role in the thinning rate, as it determines not only the sputtering yield, but also the radiolytic pathways induced by the beam and hence the type of carbonaceous structures formed in the irradiated films. Depending on the type of beam and polymer, we observe a trend for the thinning efficiency to become larger in very thin films. PMMA irradiated with 2 MeV H^+^ shows the most systematic correlation between initial thickness and thinning cross sections, while in PVC films the initial thickness plays a minor role.

## Figures and Tables

**Table 1:** Irradiation conditions of PMMA and PVC films. (dE/dx)_e_ and (dE/dx)_n_ are the electronic and nuclear stopping powers, respectively. R_p_ is the projected range of the ions, ϕ is the ion fluence, and $\phi^{*}$ is the mean deposited energy density, $\phi^{*}=\phi \times {\left(dE/dx\right)}_{e}$. The electronic and nuclear stopping power values as well as the projected range were estimated using the simulation code SRIM 2012 [25].

Ion	Polymer	(dE/dx)e [eV/nm]	(dE/dx)_n_ [eV/nm]	R_p_ [µm]	ϕ (ions/cm^2^)	$\phi^{*}$ (eV/nm^3^)
2 MeV H^+^	PVC	22.8	0.015	53.3	10^14^ to 2.8 × 10^15^	23 to 640
2 MeV H^+^	PMMA	19.2	0.013	64.2	10^14^ to 2.5 × 10^15^	19 to 480
12 MeV C	PMMA	871	0.79	13.7	10^12^ to 5 × 10^13^	9 to 435
16 MeV Au	PVC	2468	691	5.23	10^10^ to 5 × 10^13^	0.2 to 1230
17 MeV Au	PMMA	2270	561	6.07	10^11^ to 5 × 10^13^	2.3 to 1130
2.2 GeV Bi	PMMA	14,010	13.9	159	10^10^ to 7 × 10^11^	1.4 to 98

**Table 2:** PMMA thinning cross sections for different beams and initial film thicknesses.

2.1 GeV Bi		17 MeV Au		12 MeV C		2 MeV H^+^	
$h_{0}$ (nm)	σ (10^−12^) [cm^2^]	$h_{0}$ (nm)	σ (10^−14^) [cm^2^]	$h_{0}$ (nm)	σ (10^−14^) [cm^2^]	$h_{0}$ (nm)	σ (10^−16^) [cm^2^]
190	2.3 ± 0.5	800	6.0 ± 0.8	250	0.9 ± 0.5	360	3.4 ± 0.2
100	2.2 ± 0.4	400	5.5 ± 0.7	120	1.1 ± 0.5	116	5.7 ± 0.5
50	2.4 ± 0.5	200	8.2 ± 1.3	60	1.2 ± 0.4	74	8.0 ± 1.5
22	2.2 ± 0.5	90	5.8 ± 0.9	45	1.4 ± 0.3	55	7.1 ± 0.7
13	2.3 ± 1.0	40	5.6 ± 0.7	15	1.1 ± 0.3	40	6.6 ± 0.8
-	-	25	12.5 ± 0.3	-	-	20	10.4 ± 0.8
-	-	-	-	-	-	14	16.2 ± 1.1

**Table 3:** PVC thinning cross sections for different beams and initial film thicknesses.

16 MeV Au		2 MeV H^+^	
$h_{0}$ (nm)	σ (10^−14^) [cm^2^]	$h_{0}$ (nm)	σ (10^−16^) [cm^2^]
290	7.5 ± 1.8	290	1.5 ± 0.3
50	7.2 ± 1.0	92	1.3 ± 0.2
32	6.9 ± 1.8	50	1.2 ± 0.3
25	8.8 ± 0.9	30	1.1 ± 0.2
15	9.7 ± 1.1	25	1.2 ± 0.6
		15	0.8 ± 0.3

## Data Availability

The data presented in this study are available on request from the corresponding author.

## References

[1] Schrempel F., Kim Y.S., Witthuhn W. (2002). Deep ion beam lithography in PMMA: Irradiation effects. Appl. Surf. Sci..

[2] Schrempel F., Witthuhn W. (1997). Deep light ion lithography in PMMA—A parameter study. Nucl. Instrum. Methods Phys. Res. B.

[3] Ruck D.M., Schulz J., Deusch N. (1997). Ion irradiation induced chemical changes of polymers used for optical applications. Nucl. Instrum. Methods Phys. Res. B.

[4] Lee E.H. (1999). Ion-beam modification of polymeric materials—Fundamental principles and applications. Nucl. Instrum. Methods Phys. Res. B.

[5] Sum T.C., Bettiol A.A., Florea C., Watt E. (2006). Proton-beam writing of poly-methylmethacrylate buried channel waveguides. J. Light. Technol..

[6] Szilasi S.Z., Kokavecz J., Huszank R., Rajta I. (2011). Compaction of poly(dimethylsiloxane) (PDMS) due to proton beam irradiation. Appl. Surf. Sci..

[7] Yu J., Tao X., Tam H., Demokan M.S. (2005). Modulation of refractive index and thickness of poly(methyl methacrylate) thin films with UV irradiation and heat treatment. Appl. Surf. Sci..

[8] Ruck D.M. (2000). Ion induced modification of polymers at energies between 100 keV and 1 GeV applied for optical waveguides and improved metal adhesion. Nucl. Instrum. Methods Phys. Res. B.

[9] Hong W., Woo H.J., Choi H.W., Kim Y.S., Kim G.D. (2001). Optical property modification of PMMA by ion-beam implantation. Appl. Surf. Sci..

[10] Wochnowski C., Metev S., Sepold G. (2000). UV-laser-assisted modification of the optical properties of polymethylmethacrylate. Appl. Surf. Sci..

[11] Sum T.C., Bettiol A.A., van Kan J.A., Watt F., Pun E.Y.B., Tung K.K. (2003). Proton beam writing of low-loss polymer optical waveguides. Appl. Phys. Lett..

[12] Licciardello A., Fragalà M.E., Compagnini G., Puglisi O. (1997). Cross section of ion polymer interaction used to individuate single track regime. Nucl. Instrum. Methods Phys. Res. B.

[13] Rück D.M., Brunner S., Frank W., Kulisch J., Franke H. (1992). Optical waveguides in polymeric material by ion implantation. Surf. Coat. Technol..

[14] Rickards J., Zironi E.P. (1991). Chlorine loss from polyvinyl chloride under proton bombardment. Nucl. Instrum. Methods Phys. Res. B.

[15] Cota L., Adem E., Yacamán M.J. (1986). Interaction of an electron beam with a polymer surface: Study of polyvinyl chloride (PVC) using auger electron spectroscopy. Appl. Surf. Sci..

[16] Adem E., Avalos-Borja M., Rickards J., Trejo-Luna R. (1996). Microcrystals formed in proton bombarded poly(vinyl chloride) films. Radiat. Phys. Chem..

[17] Chakraborty R.N., Srivastava A.K., Singh B.K., Pathak R., Chaturvedi U.K., Nigam A.K. (1991). Effect of HCl vapours due to PVC, on PVDF samples during 250 keV H+ ion implantation. Nucl. Instrum. Methods Phys. Res. B.

[18] Davenas J., Tran V.H., Boiteux G. Ion beam induced conversion of PVC into a conducting polyene. Proceedings of the International Conference on Science and Technology of Synthetic Metals.

[19] Venkatesan T., Forrest S.R., Kaplan M.L., Murray C.A., Schmidt P.H., Wilkens B.J. (1983). Ion-beam-induced conductivity in polymer films. J. Appl. Phys..

[20] Thomaz R., Louette P., Hoff G., Muller S., Pireaux J.J., Trautmann C., Papaléo R.M. (2018). Bond-Breaking Efficiency of High-Energy Ions in Ultrathin Polymer Films. Phys. Rev. Lett..

[21] Rickards J., Trejo-Luna R., Andrade E. (1995). PVC film behavior under proton bombardment. Radiat. Phys. Chem..

[22] Davenas J., Thevenard P., Boiteux G., Fallavier M., Lu X.L. (1990). Hydrogenated carbon layers produced by ion beam irradiation of PMMA and polystyrene films. Nucl. Instrum. Methods Phys. Res. B.

[23] Zaporojtchenko V., Zekonyte J., Erichsen J., Faupel F. (2003). Etching rate and structural modification of polymer films during low energy ion irradiation. Nucl. Instrum. Methods Phys. Res. B.

[24] Zekonyte J., Zaporojtchenko V., Faupel F. (2005). Investigation of the drastic change in the sputter rate of polymers at low ion fluence. Nucl. Instrum. Methods Phys. Res. B.

[25] Ziegler J.F., Ziegler M.D., Biersack J.P. (2010). SRIM—The stopping and range of ions in matter (2010). Nucl. Instrum. Methods Phys. Res. B.

[26] Papaléo R.M., Thomaz R., Gutierres L.I., de Menezes V.M., Severin D., Trautmann C., Tramontina D., Bringa E.M., Grande P.L. (2015). Confinement Effects of Ion Tracks in Ultrathin Polymer Films. Phys. Rev. Lett..

[27] Choi H.W., Woo H.J., Hong W., Kim J.K., Lee S.K., Eum C.H. (2001). Structural modification of poly(methyl methacrylate) by proton irradiation. Appl. Surf. Sci..

[28] Thomaz R., Gutierres L.I., Morais J., Louette P., Severin D., Trautmann C., Pireaux J.J., Papaléo R.M. (2015). Oxygen loss induced by swift heavy ions of low and high dE/dx in PMMA thin films. Nucl. Instrum. Methods Phys. Res. B.

[29] Bringa E.M., Johnson R.E., Papaleo R.M. (2002). Crater formation by single ions in the electronic stopping regime: Comparison of molecular dynamics simulations with experiments on organic films. Phys. Rev. B.

[30] Sum T.C., Bettiol A.A., Seng H.L., Rajta I., van Kan J.A., Watt F. (2003). Proton beam writing of passive waveguides in PMMA. Nucl. Instrum. Methods Phys. Res. B.

[31] Choi J.O., Moore J.A., Corelli J.C., Silverman J.P., Bakhru H. (1988). Degradation of poly(methylmethacrylate) by deep ultraviolet, x-ray, electron beam, and proton beam irradiations. J. Vac. Sci. Technol. B.

[32] Kaczmarek H., Chaberska H. (2006). The influence of UV- irradiation and support type on surface properties of poly(methyl methacrylate) thin films. Appl. Surf. Sci..

[33] Ventura A., Ngono-Ravache Y., Marie H., Levavasseur-Marie D., Legay R., Dauvois V., Chenal T., Visseaux M., Balanzat E. (2016). Hydrogen Emission and Macromolecular Radiation-Induced Defects in Polyethylene Irradiated under an Inert Atmosphere: The Role of Energy Transfers toward trans-Vinylene Unsaturations. J. Phys. Chem. B.

[34] Ferry M., Ngono Y. (2021). Energy transfer in polymers submitted to ionizing radiation: A review. Radiat. Phys. Chem..

